# Mechanistic evaluation of a traditional herbal decoction in attenuating hepatic fibrosis via Nrf2/GPX4 pathway activation and ferroptosis inhibition

**DOI:** 10.1186/s41065-025-00471-y

**Published:** 2025-06-09

**Authors:** Jing-Jing Liu, Xiao-qi Zhou, Jia-Lin Zhou, Li-Jun Tong, Ling-Xiang Hu, Xiang Liu, Li-Mei Hu, Chang-Xiao Zhou, Qi Dai

**Affiliations:** 1 Jiangxi University of Chinese Medicine, Nanchang, China; 2 Guangzhou University of Chinese Medicine, Guangzhou, China; 3 Guangzhou University of Chinese Medicine-Shenzhen Hospital, Shenzhen, China; 4 Department of Gastroenterology, Affiliated Hospital of Traditional Chinese Medicine, Jiangxi University of Chinese Medicine, No. 445 of Bayi Road, Donghu District, Nanchang, 330063 China

**Keywords:** Autophagy, Blood circulation, Ferroptosis, Hepatic fibrosis, Herbal decoction promoting qi, NRF2/GPX4, Water excretion

## Abstract

**Background:**

Hepatic fibrosis, a progressive fibrotic response to chronic liver injury, is characterized by excessive collagen deposition and impaired tissue repair. This pathological process leads to liver dysfunction and potential progression to irreversible cirrhosis or hepatocellular carcinoma. Currently, therapeutic options targeting the underlying mechanisms remain limited. Traditional Chinese medicine (TCM), particularly herbal decoctions, have demonstrated efficacy in the treatment of hepatic fibrosis, although the precise mechanisms remain insufficiently elucidated.

**Objective:**

The objective of this study is to examine the mechanistic role of a TCM herbal decoction designed to promote Qi, blood circulation, and water excretion, in modulating the Nrf2/GPX4 signaling pathway and inhibiting ferroptosis in a rat model of hepatic fibrosis.

**Methods:**

A total of 17 Sprague-Dawley rats were divided into five groups. The blank control group (Group A) comprised three rats. Hepatic fibrosis was induced in the remaining rats, which were then randomized into four groups: the untreated fibrosis (Group B), TCM-treated (Group C), TCM combined with ferroptosis inhibitor (Fer-1) (Group D), and TCM combined with Fer-1 and autophagy inhibitor (3-MA) (Group E). Groups A and B received equal volumes of normal saline. Serum and hepatic tissues were collected for analysis. Serum levels of aspartate transaminase (AST), alanine aminotransferase (ALT), tumor necrosis factor-alpha (TNF-α), malondialdehyde (MDA), and iron were measured. Liver tissues were subjected to hematoxylin and eosin staining and Masson’s trichrome staining to assess pathological changes. Protein expression levels of solute carrier family 7 member 11 (SLC7A11), nuclear factor erythroid 2-related factor 2 (Nrf2), and glutathione peroxidase 4 (GPX4) were assessed using western blot analysis.

**Results:**

Group B exhibited significant deterioration compared to the control group (*p* < 0.05), including marked hepatic lipidosis and fibrosis surrounding the hepatic portal vein. Elevated levels of AST, ALT, Fe2+, MDA, TNF-α, and collagen volume were observed (*p* < 0.05), along with significantly reduced expression of GPX4, Nrf2, and SLC7A11 (*p* < 0.05). In contrast, Groups C, D, and E demonstrated significantly decreased levels of AST, ALT, Fe2+, MDA, TNF-α, and collagen volume (*p* < 0.05), accompanied by increased expression of GPX4, Nrf2, and SLC7A11 (*p* < 0.05) when compared to Group B.

**Conclusion:**

The herbal decoction demonstrated anti-fibrotic effects in a rat model of hepatic fibrosis, potentially through activation of the Nrf2/GPX4 signaling pathway and suppression of ferroptosis. These findings suggest a mechanistic basis for the observed efficacy of this TCM formulation and support its potential as a therapeutic candidate for hepatic fibrosis.

**Supplementary Information:**

The online version contains supplementary material available at 10.1186/s41065-025-00471-y.

## Background

Hepatic stellate cells become excessively activated in response to chronic liver impairment caused by various factors. This activation leads to the substantial accumulation of extracellular matrix, functioning as a reparative response to liver injury. Consequently, the normal liver architecture is disrupted, resulting in the development of hepatic fibrosis (HF) [1]. In the absence of timely and effective intervention, hepatic fibrosis may progressively deteriorate, ultimately leading to irreversible hepatic cirrhosis, and, in severe cases, hepatocellular carcinoma [2]. Therefore, implementing early therapeutic strategies to counteract hepatic fibrosis and prevent its progression to advanced stages is of paramount importance.

Traditional Chinese medicine (TCM) offers distinctive advantages in the management of hepatic fibrosis. TCM has been extensively used in both the prevention and treatment of liver diseases, with its efficacy being widely recognized. In the context of hepatic fibrosis, TCM is notable for its multifaceted therapeutic approaches, targeting the condition at various levels and through multiple mechanisms. Among these approaches, the herbal decoction promoting qi, blood circulation, and water excretion, developed by Professor Chen Kunshan based on clinical experience, has been used for the treatment of hepatic fibrosis and hepatic cirrhosis [3]. This formulation has demonstrated efficacy in suppressing the activity of the Necdin-Wnt/β-catenin signaling pathway and reducing the inhibitory effects of Wnt/β-catenin on peroxisome proliferator-activated receptor gamma, thereby impeding the progression of hepatic fibrosis [4, 5]. However, the precise mechanisms through which this herbal decoction mitigates hepatic fibrosis remain to be fully elucidated.

Ferroptosis, first detected in 2012, is an iron-dependent form of regulated cell death [6]. This process primarily results from the excessive accumulation of intracellular lipid reactive oxygen species and iron-dependent depletion or functional inactivation of glutathione peroxidase 4 (GPX4) [7]. The fundamental mechanisms underlying ferroptosis involve increased iron accumulation, impairment of the lipid repair system, and lipid peroxidation, ultimately leading to membrane disruption and apoptosis [8]. 

Aberrant iron metabolism remains a critical risk factor in the pathological progression from hepatic fibrosis to cirrhosis. Disrupted iron homeostasis is associated with metabolic dysregulation within hepatic tissues and the development of fibrosis [9]. Experimental studies in animal models have demonstrated the prominent role of transferrin in the progression of hepatic fibrosis in mice, particularly when induced by a diet high in iron content and exposure to carbon tetrachloride (CCl4). Furthermore, successful inhibition of ferroptosis ameliorates these pathological changes [10]. 

The Nrf2/GPX4 signaling pathway functions as a key antioxidant defense mechanism and is involved in the regulation of ferroptosis. Current studies have indicated that activation of the Nrf2/GPX4 signaling pathway can reduce iron accumulation, thereby mitigating cellular damage and apoptosis associated with ferroptosis. Specifically, activation of Nrf2 and upregulation of GPX4 levels attenuate intracellular oxidative stress and inhibit ferroptosis in cardiomyocytes exposed to RSL3, a known ferroptosis inducer [11]. In animal models of non-alcoholic fatty liver disease (NAFLD), metformin increases the expression of Nrf2, heme oxygenase-1, and GPX4. In contrast, the administration of brusatol, an Nrf2 inhibitor, significantly reverses the positive regulatory effects of metformin on the Nrf2/GPX4 signaling pathway and reduces its hepatoprotective effects in NAFLD models. These findings indicate that metformin may exert its liver-protective properties by activating the Nrf2/GPX4 signaling pathway and suppressing ferroptosis [12]. Therefore, activation of this pathway plays a key role in ferroptosis inhibition.

Preliminary theoretical investigations used network pharmacology and data mining techniques to examine the therapeutic potential and mechanisms of TCM in regulating ferroptosis for the treatment of HF. Initial validation was conducted using molecular docking technology, indicating that Milkvetch Root (*Radix Astragali seu Hedysari* in Latin, Huang Qi in Pinyin), Salvia Root Radix (*Radix Salviae Miltiorrhizae* in Latin, Dan Shen in Pinyin), and Suberect Spatholobus Stem (*Caulis Spatholobi* in Latin, Ji Xue Teng in Pinyin) are key components involved in modulating ferroptosis and ameliorating hepatic fibrosis. Among these, Milkvetch Root and Salvia Root Radix constitute the primary ingredient combination of the herbal decoction promoting qi, blood circulation, and water excretion.

From these theoretical findings, this study aimed to elucidate the mechanisms by which the herbal decoction promoting qi, blood circulation, and water excretion mitigates HF. The study focused on assessing the biological effects of ferroptosis on hepatocytes in a rat model of HF. Additionally, the influence of the herbal decoction on HF progression, as well as the signaling pathways and molecular targets involved was assessed. The objective of this study is to provide mechanistic insights that may guide the clinical application of this TCM formulation.

## Material

### Study animals

A total of 17 healthy male Sprague-Dawley rats of Specific Pathogen Free grade, each weighing between 200 and 220 g, were used in this study. Additional rats were made available if necessary. The animals were obtained from Beijing HFK Bioscience Co., Ltd. (License No.: SCXK [Beijing]2019–0008). The breeding environment was maintained at a temperature of 20–26 °C, with humidity levels controlled between 40% and 70%. The rats were allowed unrestricted access to food and water. Acclimatization was conducted for one week prior to the initiation of experimental procedures.

### Pharmaceuticals and reagents

The herbal decoction promoting qi, blood circulation, and water excretion was formulated using the following Chinese herbal ingredients: Milkvetch Root, White Atractylodes Rhizome (*Rhizoma Atractylodis Macrocephalae* in Latin, Bai Zhu in Pinyin), Poria (*Poria* in Latin, Fu Ling in Pinyin) with preserved peel, Plantain Seed (*Semen Plantaginis* in Latin, Che Qian Zi in Pinyin), dried shell of bottle gourd (*Lagenaria siceraria* [Molina] Standl in Latin, Hu Lu in Pinyin), Chinese Angelica (*Radix Angelicae Sinensis* in Latin, Dang Gui in Pinyin), powdered Sanqi (*Radix et Rhizoma Notoginseng* in Latin, San Qi or Tian Qi in Pinyin), Salvia Root, European Verbena Herb (*Herba Verbenae* in Latin, Ma Bian Cao in Pinyin), Chinese Thorowax Root (*Radix Bupleuri* in Latin, Chai Hu in Pinyin), Turmeric Root Tuber (*Radix Curcumae* in Latin, Yu Jin in Pinyin), Chuling (*Polyporus* in Latin, Zhu Ling in Pinyin), Oriental Waterplantain Rhizome (*Rhizoma Alismatis* in Latin, Ze Xie in Pinyin), dried Tangerine Peel (*Pericarpium Citri Reticulatae* in Latin, Chen Pi in Pinyin), and Suberect Spatholobus Stem. The formulation was supplied by the Affiliated Hospital of Jiangxi University of TCM.

The ferroptosis inhibitor, Fer-1, was obtained from Shanghai Yuanye Bio-Technology (Lot: J22HS189147), while the autophagy inhibitor, 3-MA, was acquired from Shanghai Yuanye Bio-Technology (Lot: J22HS188439). Xylene (Product No.: 33535) and absolute ethanol (Product No.: 32061) were procured from Xilong Scientific. Additionally, 95% ethanol (Product No.: 32061) was sourced from the same manufacturer.

## Methods

### Grouping and handling of the rats

Grouping and model preparation: With the exception of the blank control group, all remaining groups were randomly assigned to five treatment groups and administered a 50% CCL4 vegetable oil solution, as outlined in Table 1. Group A consisted of healthy rats (Control), while Group B served as the HF model group (Model). Group C received the herbal decoction promoting qi, blood circulation, and water excretion (TCM). Group D was treated with the same herbal decoction in combination with the ferroptosis inhibitor (Fer-1) (TCM + Fer-1). Group E was administered the herbal decoction along with both the Fer-1 and the autophagy inhibitor (PI3K inhibitor: 3-MA) (TCM + Fer-1 + 3-MA).

**Table 1:** Grouping of rats

Name of group	Mode of intervention
Group A	Control
Group B	Model
Group C	TCM
Group D	TCM + Fer-1
Group E	TCM + Fer-1 + 3-MA

Each group consisted of three rats, except for the control group (Group A) and the HF model group (Group B), which each included four rats. The injected solution ratio of CCL4 to olive oil was 1:1. Intraperitoneal injections were administered at a dose of 1.5 mL/kg, twice weekly, for four consecutive weeks. The control group received an equivalent volume of normal saline via intraperitoneal injection.

Three days prior to model induction, gastric gavage was initiated in Groups C, D, and E, and continued until 48 h after model completion. Gavage administration was conducted twice daily at 12-hour intervals for four consecutive weeks. The volume of the gavage solution was 2 mL per 100 g of body weight per day. In Groups A and B, gastric gavage was replaced with an equivalent volume of normal saline.

### Observation of hematoxylin and eosin (HE) and Masson’s trichrome staining

Liver tissues obtained from the rats were subjected to HE staining to assess pathological changes. Additionally, Masson’s trichrome staining was conducted to assess the presence of excessive collagen deposition within the liver tissues [13]. 

The primary reagents and dyes used included hematoxylin staining reagent (ZLI-9610, Beijing Zhong Shan-Golden Bridge Biological Technology), eosin staining reagent (G1100, Beijing Solarbio Science & Technology), and Masson’s trichrome staining reagent (G1006, Servicebio Technology).

### Tests using the fully automatic biochemistry analyzer

Serum samples, stored at -20 °C, were analyzed for aspartate transaminase (AST), alanine aminotransferase (ALT), and ferrous iron (Fe^2+^) levels. The analyses were conducted using a fully automatic biochemical analyzer (BK-600, Biobase Biodustry, Shandong), following the instructions provided by the manufacturer with the reagent kit. The reagents used included AST (Product No.: 70110, Biobase Biodustry, Shandong), ALT (Product No.: 70111, Biobase Biodustry, Shandong), and Fe2+ (Product No.: 70142, Shandong Biobase Biotechnology).

### Detection of TNF-α expression levels using ELISA

Hepatic blood samples were collected from the rats and subjected to centrifugation to isolate the serum. The expression levels of tumor necrosis factor-alpha (TNF-α) in the serum samples were measured using an enzyme-linked immunosorbent assay (ELISA) kit (E-EL-R2856c, Wuhan Elabscience Biotechnology), following the manufacturer’s instructions.

### Detection of relevant protein expression levels using western blot method

Liver tissue proteins were extracted using RIPA cell lysis buffer (C1053, Beijing Pulley Gene Technology), and their concentrations were measured using the BCA method. Equal amounts of protein samples were then prepared for western blot analysis to quantify the levels of GPX4, NRF2, and SLC7A11 proteins [14, 15]. Protein bands were recorded using ultra-high sensitivity chemiluminescence imaging system instrument (Chemi DocTM XRS+, BIO-RAD Laboratories, Shanghai). Table 2 provides information about the antibodies used and their respective dilution factors.

**Table 2:** WB antibodies

Name of the antibody	Dilution factor
Mouse Anti-B-Actin(HC201, TransGen Biotech)	1/2000
HRP conjugated Goat Anti-Mouse IgG(H + L)(GB23301, Servicebio)	1/2000
Rabbit Anti LC3 (12741, CST)	1/1000
Rabbit Anti TFR1 (AF5343, Affinity)	1/1000
Rabbit Anti NRF2 (ab92946, Abcam)	1/2000
Rabbit Anti SLC7A11 (26864-1-AP, Proteintech)	1/1000
Mouse Anti GPX4(67763-1-Ig, Proteintech)	1/2000
HRP conjugated Goat Anti-Rabbit IgG (H + L)(GB23303, Servicebio)	1/2000

### Statistical analysis

All data are expressed as the mean ± standard deviation (x̅ ± s). Statistical analyses were conducted using SPSS 20.0 software. Comparisons of numerical values among multiple groups were performed using one-way analysis of variance, with the significance level set at α = 0.05 (*p* < 0.05 was considered statistically significant). Graphs were generated using GraphPad Prism 9.0 software.

## Results

### Observation of pathological changes in rat liver tissues in each group

**Fig. 1:**
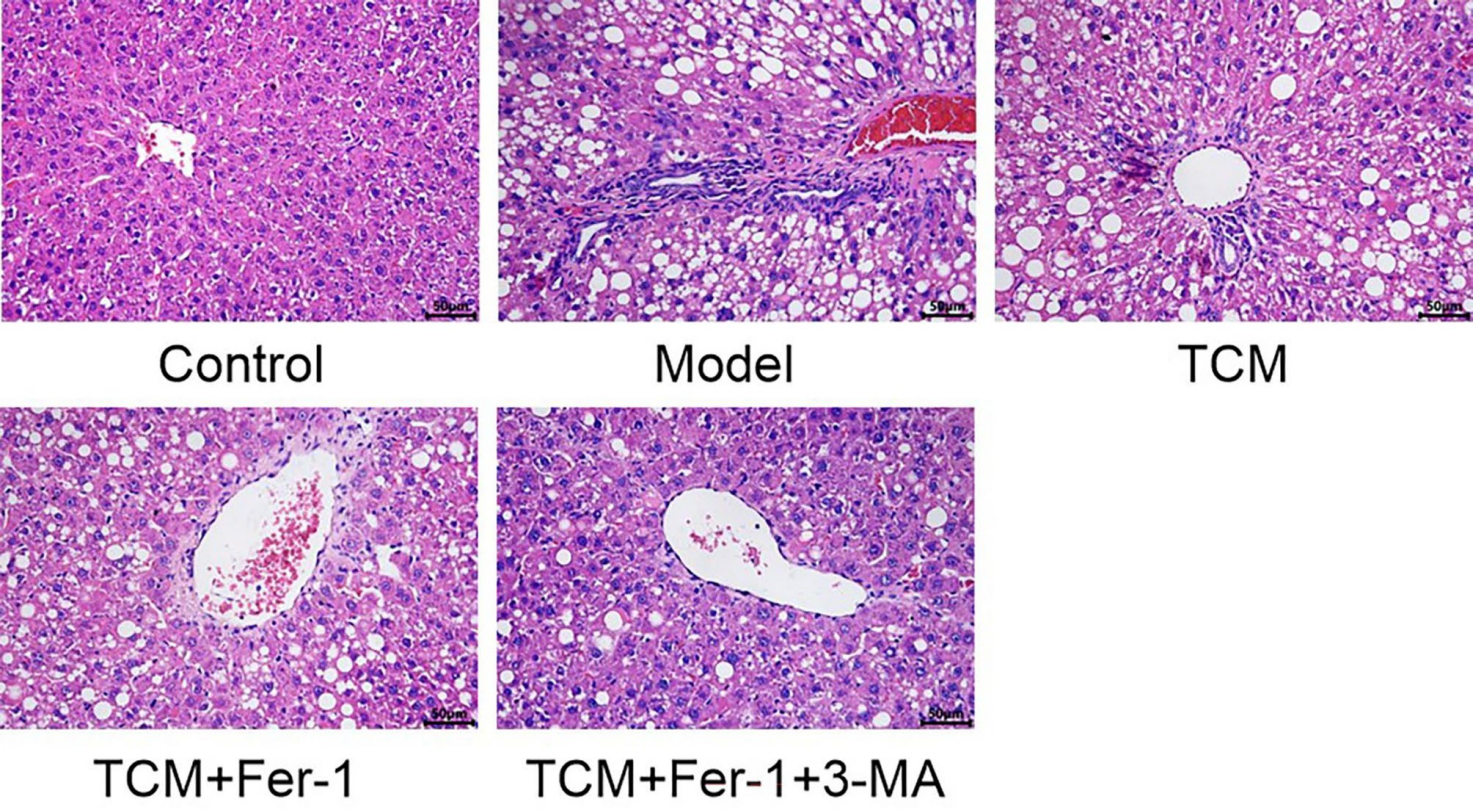
Histopathological features observed in each group (HE staining, ×400)

Figure 1 presents the results of HE staining. Liver tissues from rats in the blank control group indicated no significant pathological changes. In Group A, the hepatic lobule structure remained intact, with liver cells appearing uniform in size and hepatic cords arranged radially, without evidence of inflammatory cell infiltration or collagen deposition.

In contrast, Group B demonstrated marked fatty lesions and fibrosis formation around the portal vein when compared to the blank control group. Following the administration of the therapeutic intervention, Groups C, D, and E exhibited a significant reduction in hepatic fibrosis compared to Group B [16]. 

The degree of inflammatory activity and liver tissue fibrosis was assessed using the METAVIR scoring system, as detailed in Table 3 [17]. 

**Table 3:** Pathological score

Group	Inflammation score	Fibrosis score
Control	0.00 ± 0.00	F0
Model	8.33 ± 0.33^*^	F3
TCM	6.00 ± 0.58^#^	F2
TCM + Fer-1	5.00 ± 0.58^#^	F2
TCM + Fer-1 + 3-MA	3.67 ± 0.33^#^	F1

Note: *: Compared with control group, *P* < 0.05; #: Compared with the model group, *P* < 0.05

### Observation of HF using Masson’s trichrome staining

**Fig. 2:**
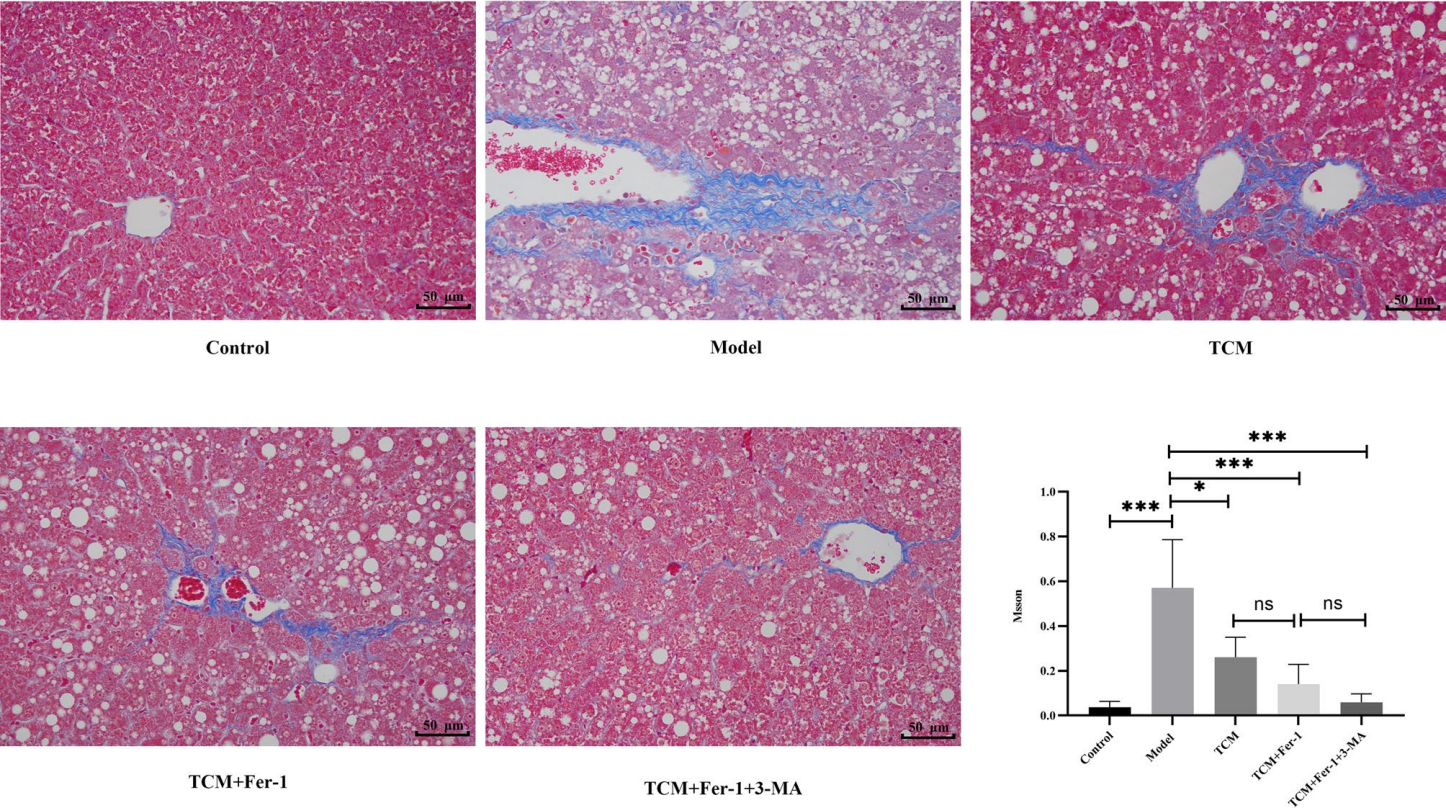
Hepatic fibrosis conditions demonstrated in each group (Masson staining, ×400)

The experimental results are presented in Fig. 2; Table 4. A significantly higher collagen volume was observed in the liver tissue of Group B compared to the blank control group (*p* < 0.05). In contrast, Groups C, D, and E demonstrated a significant reduction in collagen volume when compared to Group B (*p* < 0.05).

**Table 4:** Quantitative assessment of Masson’s trichrome staining in each group

Group	Masson
Control	0.04 ± 0.03
Model	0.57 ± 0.22^*^
TCM	0.26 ± 0.09^#^
TCM + Fer-1	0.09 ± 0.01^#^
TCM + Fer-1 + 3-MA	0.06 ± 0.04^#^

Note: *: Compared with control group, *P* < 0.05; #: Compared with the model group, *P* < 0.05

### Biochemical and ELISA test results

**Fig. 3:**
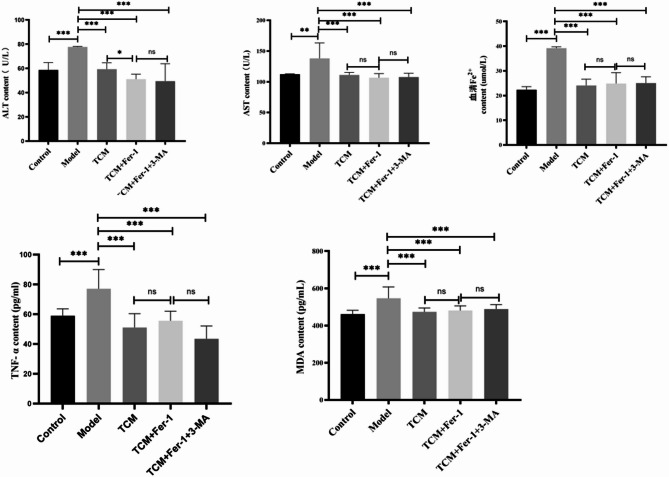
Serum biochemical parameters and ELISA results. Statistical significance is indicated as follows: * *p* < 0.05, ** *p* < 0.01, ***p < 0.001

The levels of ALT, AST, Fe^2+^, TNF-α, and malondialdehyde (MDA) in rat serum were measured using a fully automatic biochemical analyzer and ELISA kits. The results are presented in Fig. 3.

A significant increase in serum levels of ALT, AST, Fe^2+^, TNF-α, and MDA was observed in Group B compared to the blank control group (*p* < 0.05). In contrast, Groups C, D, and E demonstrated decreased levels of these biomarkers in serum when compared to Group B (*p* < 0.05).

### Expression levels of GPX4, NRF2, and SLC7A11 proteins in rat liver tissue detected using WB

**Fig. 4:**
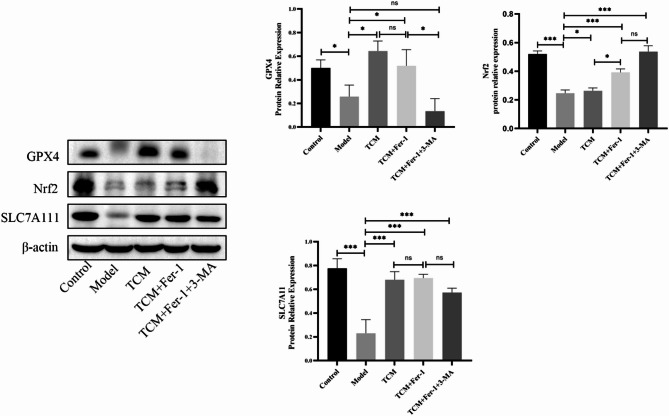
Expression levels of proteins associated with ferroptosis. Statistical significance is indicated as follows: * *p* < 0.05, ** *p* < 0.01, *** *p* < 0.001

The results presented in Fig. 4 demonstrate significant differences among the groups. Group B exhibited a marked reduction in the expression levels of GPX4 and SLC7A11 proteins compared to the blank control group (*p* < 0.05), along with a slight decrease in NRF2 protein expression.

In contrast, Groups C, D, and E indicated significant increases in SLC7A11 protein levels compared to Group B (*p* < 0.05), while a slight elevation in NRF2 protein expression was also observed. Furthermore, a significant increase in GPX4 protein expression was detected in Groups C and D (*p* < 0.05). In Group E, GPX4 protein expression was decreased; however, the difference did not reach statistical significance (*p* > 0.05).

## Discussion

HF represents a critical pathological process in the progression of various chronic liver diseases toward cirrhosis or hepatocellular carcinoma, serving as a common pathway in liver injury [16]. CCl4 is widely used as an inducer of liver fibrosis due to its operational simplicity and high similarity to human hepatic fibrosis [18]. The administration of CCl4 in rats induces sustained hepatic injury through recurrent and persistent necrosis of hepatocytes, ultimately leading to hepatic fibrosis.

In this study, significant pathological changes were observed in the liver tissues of rats in Group B following repeated administration of low-dose CCl4, administered twice weekly for four consecutive weeks. These findings confirmed the successful establishment of the hepatic fibrosis model. Serum levels of AST and ALT are widely recognized as sensitive biomarkers for assessing liver function impairment [19]. 

Experimental data indicated a significant increase in the activities of AST and ALT enzymes in Group B compared to the control group, indicating substantial liver damage. In contrast, Groups C, D, and E demonstrated significantly lower serum levels of AST and ALT compared to Group B, indicating that the herbal decoction promoting qi, blood circulation, and water excretion exerts a protective effect in rats with hepatic fibrosis (Fig. 3; Table 5).

**Table 5:** Serum biochemical index

Group	ALT(u/L)	AST(u/L)	Fe^2+^(u/L)	TNF-α (pg/mL)	MDA (pg/mL)
Control	58.77 ± 6.01	112.20 ± 0.66	22.35 ± 1.27	58.93 ± 4.67	461.84 ± 20.13
Model	77.69 ± 0.48^*^	138.07 ± 25.17^*^	39.19 ± 0.55^*^	77.05 ± 12.94^*^	546.46 ± 61.46^*^
TCM	59.32 ± 5.28^#^	111.02 ± 4.22^#^	24.05 ± 2.59^#^	51.04 ± 9.32^#^	473.48 ± 21.15^#^
TCM + Fer-1	51.14 ± 4.09^#^	106.57 ± 6.70^#^	24.86 ± 4.39^#^	55.61 ± 6.41^#^	480.54 ± 25.37^#^
TCM + Fer-1 + 3-MA	49.47 ± 14.46^#^	107.59 ± 6.40^#^	25.04 ± 2.59^#^	43.41 ± 8.66^#^	488.44 ± 23.85^#^

Note: *: Compared with control group, *P* < 0.05; #: Compared with the model group, *P* < 0.05

Oxidative stress and inflammatory responses are pivotal in the pathogenesis and progression of hepatic fibrosis. An imbalance in the regulation of oxidative free radicals can lead to their excessive generation, resulting in an abnormal elevation of MDA levels, a classical end product of lipid peroxidation [20]. According to research, it was indicated that ROS may contribute to the initiation and progression of fibrosis, implying that oxidative stress may exacerbate fibrotic changes [21–23]. 

The assessment of serum MDA levels is used as an indicator of oxidative damage, while the measurement of TNF-α levels in serum serves as a marker of inflammatory response.

Nuclear factor erythroid 2-related factor 2 (Nrf2) serves as a key regulatory factor within the cellular defense system against oxidative stress [24]. Studies have demonstrated that mulberry extract activates the Nrf2 signaling pathway, thereby promoting the synthesis of antioxidant enzymes. This activation significantly suppresses oxidative stress, ferroptosis, and hepatic inflammation, indicating its potential anti-fibrotic effects [25]. 

The Ginseng, Poria, and White Atractylodes Rhizome Powder formula has demonstrated therapeutic efficacy in reducing oxidative stress through the upregulation of the Nrf2 signaling pathway. This enhancement of Nrf2 expression effectively inhibits ferroptosis in hepatocytes, contributing to the alleviation of alcoholic liver injury. In addition, the formula exhibits both antioxidant and anti-inflammatory properties, further supporting its potential as a therapeutic intervention [26]. 

GPX4 is a monomeric glutathione peroxidase enzyme that plays a key role in reducing hydroperoxides within lipid membranes. This function establishes GPX4 as an essential component of the enzymatic defense system against lipid peroxidation [27]. 

Deficiency in GPX4 induces ferroptosis, a process characterized by the inability of GPX4 to adequately degrade phospholipid hydroperoxides, such as arachidonic acid hydroperoxide. This deficiency leads to the accumulation of lipid peroxides, ultimately initiating ferroptosis [28, 29]. 

Research has shown that irisin inhibits ferroptosis in hippocampal neurons by modulating the Nrf2/GPX4 signaling pathway. This inhibition has been associated with improvements in the inflammatory microenvironment observed in sepsis-associated encephalopathy [30]. 

This study aimed to assess the potential therapeutic effects of an herbal decoction promoting qi, blood circulation, and water excretion in the prevention of HF. The investigation focused on elucidating the underlying mechanisms, particularly the activation of the NRF2/GPX4 signaling pathway, to inhibit hepatocytic ferroptosis in a rat model of CCL4-induced HF.

The experimental results indicated a significant reduction in the protein expression levels of NRF2 and GPX4 in Group B compared to the control group. In contrast, Groups C, D, and E displayed varying degrees of increased expression levels of NRF2 and GPX4 when compared to Group B, with the exception of Group E.

SLC7A11, the core subunit of the System Xc- complex, plays a substantial role in maintaining cellular redox balance by mediating the import of cystine, which is essential for glutathione (GSH) synthesis. Cysteine, synthesized from cystine, acts as a key antioxidant that mitigates lipid peroxidation, thereby lowering lipid peroxide levels and preventing ferroptosis [31]. 

The interaction between Beclin-1 and SLC7A11 represents a key regulatory step within the System Xc- structure. This interaction leads to the suppression of SLC7A11, resulting in the inhibition of ferroptosis, a process facilitated by phosphorylation at serine residues 90, 93, and 96 [32]. Disruption of hepatic iron metabolism can lead to iron overload, which may exacerbate ferroptosis [33, 34]. 

The experimental results demonstrated a significant decrease in the expression level of SLC7A11 protein and a significant increase in iron levels in Group B compared to the control group. In contrast, Groups C, D, and E exhibited significantly higher expression levels of SLC7A11 protein and a notable reduction in iron levels when compared to Group B. These findings indicate that the herbal decoction promoting qi, blood circulation, and water excretion effectively suppresses the onset of hepatic ferroptosis.

Emerging evidence increasingly supports the notion that ferroptosis is closely associated with autophagy, a fundamental process for maintaining cellular homeostasis. Dysregulated autophagy or enhanced lysosomal activity results in the accumulation of iron ions and lipid peroxides within cells, thereby promoting ferroptosis [35]. The autophagic degradation of ferritin facilitates the release of Fe^3+^, leading to an increase in intracellular free iron levels and subsequently triggering ferroptosis. Furthermore, suppression or deletion of nuclear receptor coactivator 4 (NCOA4) expression, along with the inhibition of autophagy, has been found to significantly reduce ferroptosis incidence [36]. 

Aryl hydrocarbon receptor nuclear translocator-like protein 1 (ARNTL1), a key protein involved in circadian rhythm regulation, undergoes selective degradation through p62-mediated autophagy. This degradation mechanism results in increased expression of Hypoxia-inducible factor hydroxylase 1 (PHD1), promoting lipid peroxidation and consequently facilitating ferroptosis [37, 38]. 

Additionally, studies have revealed that a reduction in GSH levels is associated with increased expression of the autophagy marker protein microtubule-associated protein 1 light chain 3, heightened formation of autophagic vacuoles, and pronounced activation of autophagy [33, 39]. 

However, further investigation is required to elucidate how the herbal decoction promoting qi, blood circulation, and water excretion influences ferroptosis and contributes to the improvement of liver fibrosis in rats through the modulation of autophagy.

## Conclusion

The herbal decoction promoting qi, blood circulation, and water excretion demonstrates potential in mitigating oxidative stress and inflammation, regulating iron metabolism, and preventing iron overload. These effects contribute to the inhibition of hepatocytic ferroptosis, suggesting therapeutic benefits in managing HF. The underlying mechanism involves the activation of the NRF2/GPX4 signaling pathway, which suppresses hepatocytic ferroptosis and offers protective effects in rats with HF.

However, limitations related to the small sample size and the unclear role of autophagy signaling in ferroptosis inhibition warrant further investigation. Additional studies are required to clarify these aspects and to validate the observed therapeutic effects.

## Electronic supplementary material

Below is the link to the electronic supplementary material.

Supplementary Material 1

Supplementary Material 2

Supplementary Material 3

Supplementary Material 4

## Data Availability

The datasets used and/or analyzed during the current study are available from the corresponding author upon reasonable request.

## Abbreviations

GPX4: Glutathione peroxidase 4
Nrf2: Nuclear factor erythroid 2-related factor 2
HF: Hepatic fibrosis
TCM: Traditional Chinese medicine
SD: Sprague-dawley
Fer-1: Ferrostatin-1
3-MA: 3-Methyladenine
AST: Aspartate transaminase
ALT: Alanine aminotransferase
TNF-a: Tumor necrosis factor-α
MDA: Malondialdehyde
HE: Hematoxylin and eosin staining
WB: Western blot
SLC7A11: Solute carrier family 7 member 11
ROS: Reactive oxygen species
RSL3: RAS-selective lethal 3
NAFLD: Non-alcoholic fatty liver disease
HO-1: Heme oxygenase 1
SPF: Specific pathogen-free
PI3K: Phosphoinositide 3-kinase
ELISA: Enzyme linked immunosorbent assay
RIPA: Radio immunoprecipitation assay
PL: Phospholipides
GSH: L-glutathione
NCOA4: Nuclear receptor coactivator 4
ARNTL1: Aryl hydrocarbon receptor nuclear translocation-like protein 1
PHD1: Hypoxia-inducible factor hydroxylase 1
LC3: Microtubule-associated protein 1 light chain 3
