# Supplementary figures and images for: Mechanistic evaluation of a traditional herbal decoction in attenuating hepatic fibrosis via Nrf2/GPX4 pathway activation and ferroptosis inhibition
Source: Hereditas. 2025 Jun 9;162:100. doi: 10.1186/s41065-025-00471-y (PMC12147262; doi:10.1186/s41065-025-00471-y)

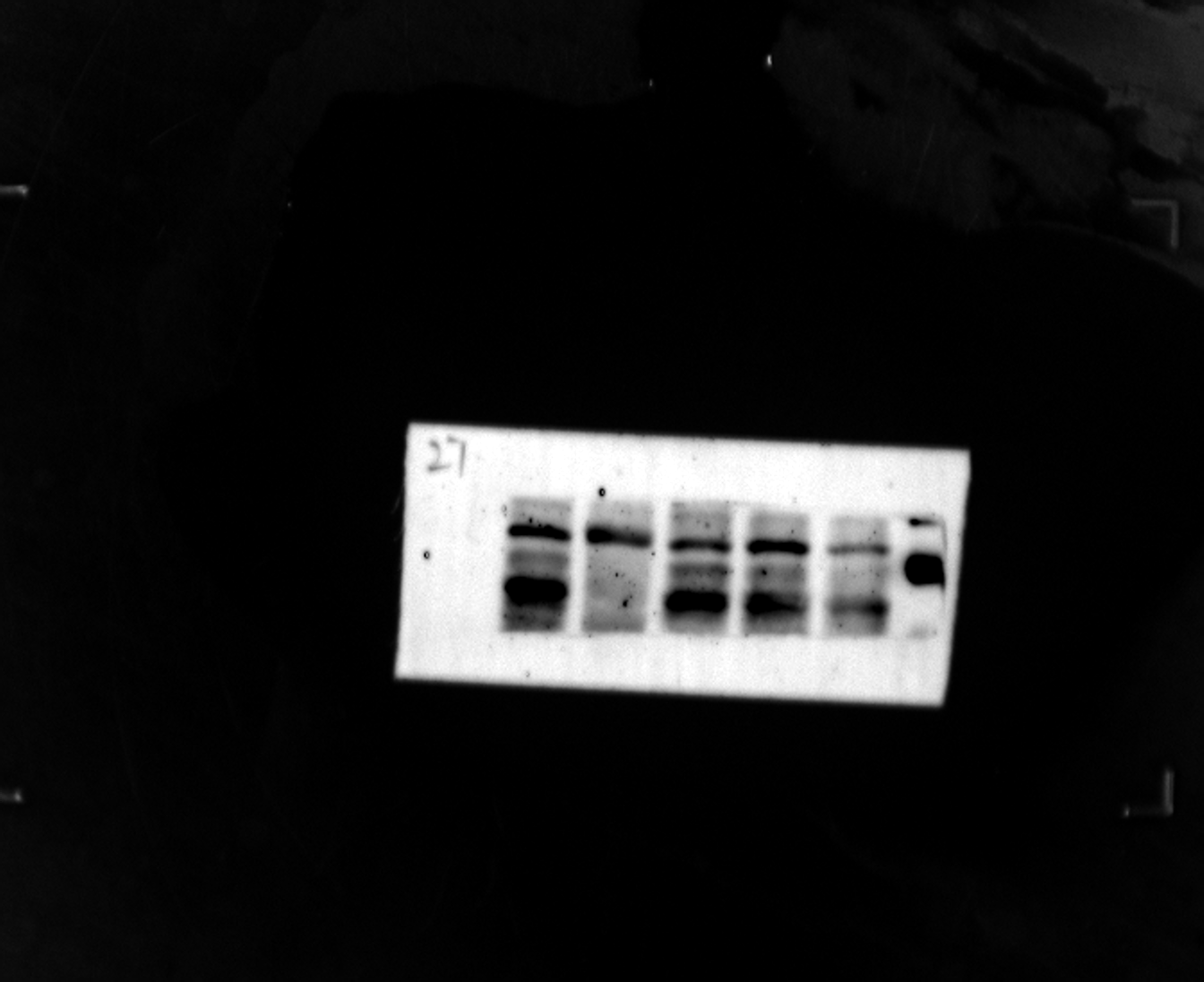

Supplement: Supplementary file 1 — Supplementary Material 1 [file 41065_2025_471_MOESM1_ESM.tif]

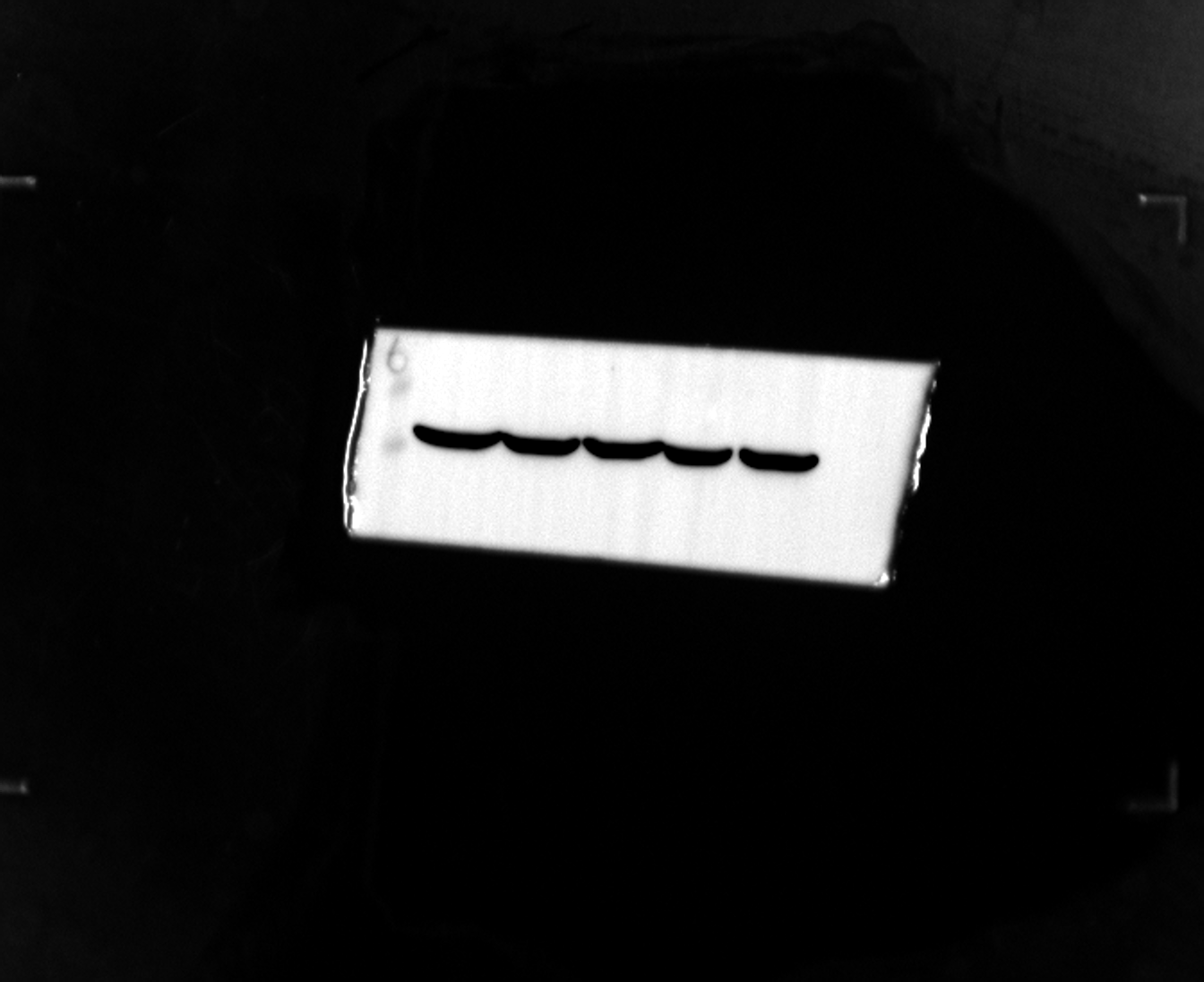

Supplement: Supplementary file 2 — Supplementary Material 2 [file 41065_2025_471_MOESM2_ESM.tif]

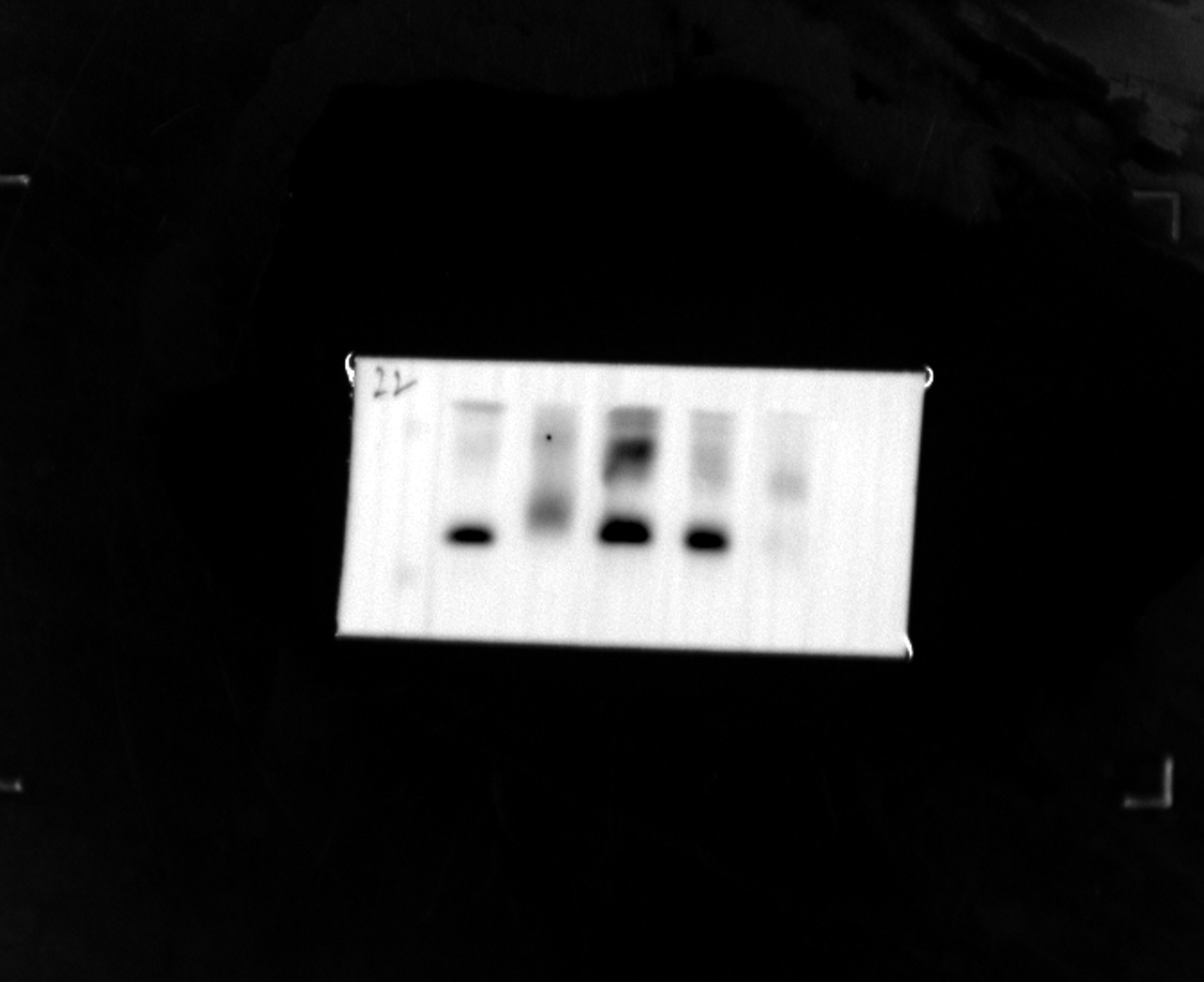

Supplement: Supplementary file 3 — Supplementary Material 3 [file 41065_2025_471_MOESM3_ESM.tif]

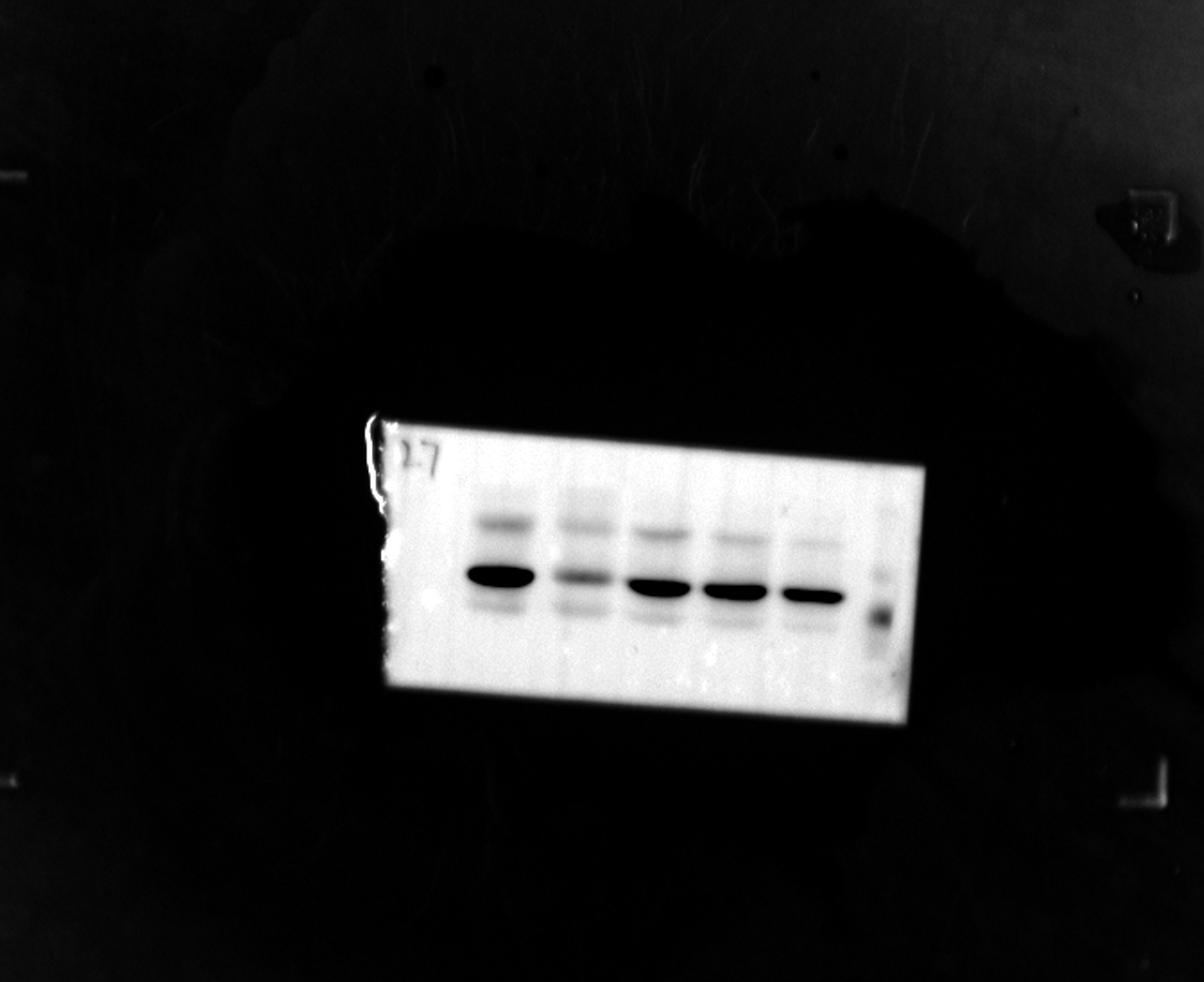

Supplement: Supplementary file 4 — Supplementary Material 4 [file 41065_2025_471_MOESM4_ESM.tif]
